# Convergence of gut phage communities but not bacterial communities following wild mouse bacteriophage transplantation into captive house mice

**DOI:** 10.1093/ismejo/wrae178

**Published:** 2024-09-14

**Authors:** Dagmar Čížková, Pavel Payne, Anna Bryjová, Ľudovít Ďureje, Jaroslav Piálek, Jakub Kreisinger

**Affiliations:** Institute of Vertebrate Biology of the Czech Academy of Sciences, Květná 8, 603 00, Brno, Czech Republic; Institute of Vertebrate Biology of the Czech Academy of Sciences, Květná 8, 603 00, Brno, Czech Republic; Department of Zoology, Faculty of Science, Charles University, Viničná 7, 128 44, Prague, Czech Republic; Institute of Vertebrate Biology of the Czech Academy of Sciences, Květná 8, 603 00, Brno, Czech Republic; Institute of Vertebrate Biology of the Czech Academy of Sciences, Květná 8, 603 00, Brno, Czech Republic; Institute of Vertebrate Biology of the Czech Academy of Sciences, Květná 8, 603 00, Brno, Czech Republic; Department of Zoology, Faculty of Science, Charles University, Viničná 7, 128 44, Prague, Czech Republic

**Keywords:** gut microbiome, bacteriome, phageome, bacteriophages, house mouse, phageome transplantation, wild, captivity, specific-pathogen-free

## Abstract

Bacteriophages are abundant components of vertebrate gut microbial communities, impacting bacteriome dynamics, evolution, and directly interacting with the superhost. However, knowledge about gut phageomes and their interaction with bacteriomes in vertebrates under natural conditions is limited to humans and non-human primates. Widely used specific-pathogen-free (SPF) mouse models of host-microbiota interactions have altered gut bacteriomes compared to wild mice, and data on phageomes from wild or other non-SPF mice are lacking. We demonstrate divergent gut phageomes and bacteriomes in wild and captive non-SPF mice, with wild mice phageomes exhibiting higher alpha-diversity and interindividual variability. In both groups, phageome and bacteriome structuring mirrored each other, correlating at the individual level. Re-analysis of previous data from phageomes of SPF mice revealed their enrichment in *Suoliviridae* crAss-like phages compared to our non-SPF mice. Disrupted bacteriomes in mouse models can be treated by transplanting healthy phageomes, but the effects of phageome transplants on healthy adult gut microbiota are still unknown. We show that experimental transplantation of phageomes from wild to captive mice did not cause major shifts in recipient phageomes. However, the convergence of recipient-to-donor phageomes confirmed that wild phages can integrate into recipient communities. The differences in the subset of integrated phages between the two recipient mouse strains illustrate the context-dependent effects of phage transplantation. The transplantation did not impact recipient gut bacteriomes. This resilience of healthy adult gut microbiomes to the intervention has implications for phage allotransplantation safety.

## Introduction

Bacterial communities play pivotal roles within vertebrate holobionts, particularly in the digestive system. Extensive research has unveiled their profound influence on the holobiont’s phenotype, encompassing metabolism [1], immunity [2], and neuro-endocrine functions [3], collectively impacting holobiont physiology and overall health [4]. Beyond its biomedical significance, the gut bacteriome’s role in vertebrate evolution, adaptation, and wildlife conservation is garnering increasing attention [5, 6].

The composition and function of the gut bacteriome are regulated by the gut environment, which includes holobiont immunity, diet, and other members of the gut ecosystem [7]. Notably, bacterial viruses, known as bacteriophages, are recognized as major modulators of bacterial population dynamics and critical factors shaping bacterial evolution [8–10].

The outcome of phage–bacteria interactions is significantly influenced by the type of phage infection. In lytic infections, phage reproduction is accompanied by host cell destruction, while in lysogenic infections, phages integrate their genomes into bacterial chromosomes and replicate as prophages alongside host bacteria. Prophages can burden the host, but can also enhance the fitness of lysogens by aiding in the exploitation of superhosts, resistance to other phages, competition with other microbes, and adaptation to adverse environmental conditions [10, 11]. Virulent phages, capable only of lytic infections, interact with host bacteria in a predominantly antagonistic manner, resembling a predator–prey relationship [8, 12]. Interactions between temperate phages and their hosts are more intricate as temperate phages can establish either lytic or lysogenic infections, and prophages can switch to the lytic cycle under certain conditions [13]. Crucially, phage-bacterium interactions occur within a broader context that includes other microbes and the superhost, whose immunity can directly respond to both bacteria and phages [14–16]. These contextual factors can significantly impact the evolution of phage–bacteria interactions, the fitness consequences of harboring prophages, and the lysis-lysogeny decision of temperate phages [17–19].

Diversity and composition of the human gut phageome change during childhood along with the gut bacteriome, but stabilize in adulthood and become highly individual-specific [20–22]. The adult human phageome can experience diet and disease-specific changes, distinct from shifts in gut bacteriomes [23–28]. However, our understanding of phageome composition and the factors determining phage communities in other vertebrate species remains largely unknown, with studies involving free-living populations primarily limited to non-human primates [29, 30].

The most common experimental model for studying the gut microbiota is the specific-pathogen-free (SPF) laboratory mouse. However, the gut bacteriome of SPF mice undergoes significant changes compared to their wild-living counterparts, resulting in impaired interactions between the bacteriome and the superhost, altered superhost phenotypes, and reduced fitness [31]. Although the phageome in laboratory mice has been examined [32], information on the phageome in wild-living mice or captive mice bred under non-SPF conditions is notably lacking. This information is crucial for assessing how gut phages relate to the dysbiosed bacteriome of laboratory mice.

Transplanting complex microbial communities has proven effective in treating certain gastrointestinal diseases in humans [33]. Some studies suggest a possible role of bacteriophages in these beneficial effects [34–36] and have even demonstrated that dysbiotic gut microbiota can be modulated by transplanting healthy phageomes [37–40]. Transplanting viral filtrates offers a potentially safer alternative to whole-microbiota transplants and can be effective against a range of gastrointestinal conditions compared to phage therapy targeting specific bacteria as disease agents [41]. However, baseline information on the effects of phageome allotransplantation on non-dysbiosed gut microbiota and their variability in different superhost and microbiota contexts is lacking.

This study aims to provide insights into phageome composition in wild house mouse populations and describe their divergence compared to captive individuals from conventional non-SPF breeding facilities. By integrating gut bacteriome profiles with phageome data, we analyze how these two components of the gut microbiota interact in captive and wild populations. These data may elucidate the role of phages in the gut microbiome of non-primate mammals and help create more realistic laboratory models that reflect coevolution between the superhost, symbiotic bacteriome, and phageome under natural conditions.

The second aim is to assess the effect of transplanting phageomes from wild-living mice into the gut microbiota of healthy adult mice in captivity. We examine transplants of identical wild phageomes in a variable context represented by two mouse strains with different gut microbiota. Our investigation focuses on posttransplantation changes in both the phageomes and bacteriomes of the recipients, with anticipation of four possible outcomes: (1) no changes due to the resistance of recipient gut bacteria to wild donor phages; (2) shift in recipient phageomes towards wild donors without changes in recipient bacteriomes, indicating that wild phages can infect captive bacteriomes but that healthy adult bacterial communities remain resilient to the altered phageomes; (3) shift in recipient phageomes and bacteriomes towards wild donors due to increased susceptibility of captive bacteria to wild phages, implying targeted regulation of the microbial community by the transplanted phageomes; and (4) significant non-directional changes in both phageomes and bacteriomes of recipients, indicating dysbiosis of microbial communities and potential health risks. This approach provides essential information on the role of phages as regulators of gut bacteriomes and establishes a baseline for the safety of medical phageome transplants.

## Materials and methods

### Experimental animals

We used 26 adult house mice, aged between 165 and 201 days, from two wild-derived inbred strains, BUSNA and BULS [42, 43]. These strains belong to the subspecies *Mus musculus musculus.* Their founders were captured in the Czech Republic and have been kept at the Institute of Vertebrate Biology, CAS, for more than 20 years. The mice are maintained within a conventional multi-purpose breeding facility without barriers and without interventions to establish and maintain a SPF state.

Ten adult wild mice of the same subspecies were live-trapped in May 2020 at three different localities (Jedov [Jed]: 49.2257072 N, 16.1563911E; Naloučany [Nlc]: 49.2346828 N, 16.1326228E; Velké Pole [Vpol]: 49.1921358 N, 16.1717658E), with distances between localities <10 km.

Care of all mice mentioned in this manuscript and all procedures and experiments were performed according to the National Institutes of Health and the Office of Laboratory Animal Welfare guidelines for appropriate animal husbandry (licenses number: 61974/2017 MZE 17214 and 62 065/2017 MZE 17214), and all live mice were handled by an authorized person.

### Transplantation of wild phageomes to captive mice

**Figure 1:**
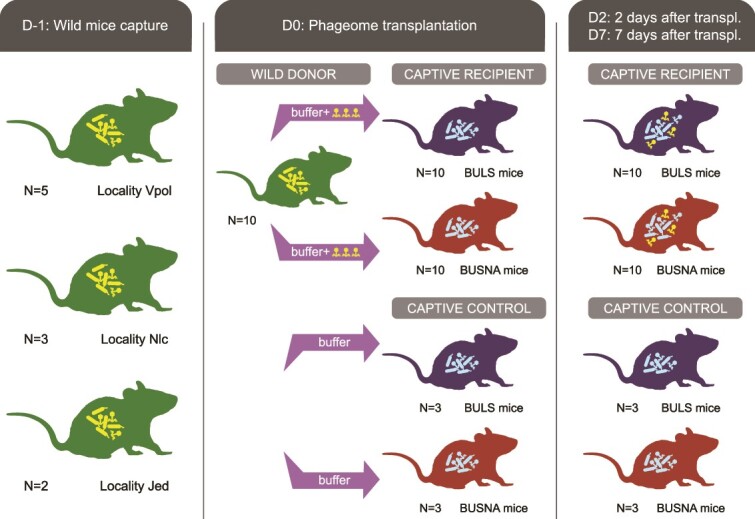
On Day D-1, 10 wild mice were captured at three localities (Vpol, Nlc, Jed). On Day D0, caecal filtrates from wild donors were transplanted to captive recipients from two wild-derived strains—BULS and BUSNA (one BULS and one BUSNA mouse per wild donor); three mice from each strain were used as non-transplanted controls; bacteriomes and phageomes from wild and captive mice were examined using fecal samples collected at D-1, D0 and 2 and 7 days posttransplantation (D2, D7).

Caecal contents from the wild mice, enriched for virus-like particles through filtration, were transplanted to captive mice. Wild mice were caged separately using sterile filter paper as bedding for ∼1 h until defecation and then euthanized. Their caecal contents were collected, resuspended in 800 μl of sterile ice-cold SMG buffer, vortexed with 2.8 mm ceramic beads at maximum speed for 30 s, and filtered through 0.45 μm and 0.22 μm Polyethersulfone (PES) syringe filters. We administered 250 μl of these filtrates to the recipient mice, which had fasted for 6 h, via oral gavage using polypropylene tubes. Phageome from each wild donor was transplanted to one BUSNA and one BULS mouse. Totally 10 wild donors infected 10 recipients from each strain, and 3 mice of each strain served as non-transplanted controls, receiving clean SMG buffer (Fig. 1, Supplementary Table S1).

### Monitoring of gut bacteriome and phageome

Composition of gut bacteriome and phageome was determined by 16S rRNA gene amplicon sequencing and metagenomic sequencing of fecal DNA, respectively. Feces from the wild donors were collected on the day of trapping (D0), and feces from the BUSNA and BULS recipients and controls were collected 1 day before the transplantation (D-1) and 2 (D2) and 7 days (D7) posttransplantation.

For phageome sequencing, we resuspended three fecal droppings in 1 ml of SMG buffer and filtered them as described earlier. As an internal control, each filtrate was spiked with T7 bacteriophage (10^6^ pfu of T7 culture per 200 μl of the fecal filtrate). We then treated the samples with Benzoase (13.5 U/ml of filtrate; 15 min at 37°C) to remove free-floating DNA, followed by Proteinase K (200 μg/ml; 5 min at 56°C) and QIAGEN AL lysis buffer (200 μl; 15 min at 56°C). Bacteriophage DNA was isolated using the QIAGEN QIAamp MinElute Virus Spin kit. Sequencing libraries were prepared as described in Glenn *et al*. [44], using the ROCHE Kapa HyperPlus kit and iTrue primers, which constrain the sequencing to dsDNA genomes. Whole-genome amplification was not performed, but the libraries were amplified during indexing polymerase chain reaction (PCR) using 13 PCR cycles, and then sequenced on one lane of a HiSeq X System (Illumina) with 150 bp paired-end reads (Novogene, UK).

Total fecal DNA was extracted using the QIAGEN DNeasy PowerSoil kit. Bacterial 16S rRNA gene amplicon sequencing libraries were constructed using a two-step PCR, each sample in a technical duplicate, as described in Čížková *et al*. [45], and sequenced using a MiSeq System (Illumina) with 300 bp paired-end reads (CEITEC Genomics Core Facility, Brno, Czechia).

### Bioinformatics

Bacterial 16S rRNA gene amplicon sequencing data were processed into Amplicon Sequence Variants (ASVs) using dada2 [46] following the pipeline described in Čížková *et al*. [45]. Metagenomic sequences were assembled with metaSPAdes [47] both collectively to create a meta-reference and for individual samples. Contigs identified as viral/phage by at least one of the three approaches were preselected: Marvel [48] (contigs ≥4 kb), Cenote-Taker [49] (contigs ≥3 kb), and BlobTools [50] (contigs ≥0.5 kb). The taxonomy of these contigs was assigned using Demovir (https://github.com/feargalr/Demovir) based on DIAMOND [51] searches against the Viruses subset of the UniProt reference proteomes database. The complete T7 genome (i.e. spiked-in control) was recovered in each sample and in the meta-reference, verifying the laboratory and bioinformatic procedures. Non-phage contigs and T7 contigs were excluded. The abundance of phage contigs in the samples was estimated by mapping reads to the meta-reference. CheckV was used to estimate the completeness of phage genomes [52]. Due to uncertainty regarding the CheckV estimates (results are presented in Supplementary Methods), the lifestyle of the phages (virulent vs. temperate) was assessed using PhaTYP, which has high accuracy with low dependence on genome completeness [53]. Bacterial hosts of the phages were predicted using iPHoP software [54].

Phageome sequences from six samples (ERR2059953-ERR2059956, ERR2059965-ERR2059967) of SPF laboratory mice generated by Kim and Bae [32] were downloaded from European Nucleotide Archive (ENA) database and analyzed using the bioinformatics pipeline described above.

The bioinformatic analyses are described in more detail in Supplementary Methods.

### Statistics

Variation in alpha diversity of phageomes (phage contigs) and bacteriomes (ASVs) among mouse groups and treatment levels was assessed using linear (mixed-effect) models employing the Shannon index and community richness.

For the analysis of bacteriome composition, we utilized Bray–Curtis (reflecting ASV relative abundance) and Jaccard (reflecting ASV presence/absence) dissimilarities between samples. Similarly, phageome composition was analyzed using Bray–Curtis and Jaccard dissimilarities, both based on the counts of reads mapped to phage contigs from the meta-reference. Analogous dissimilarities (i.e. probWJ and Jexact) between the sequence content of phageomes were calculated for separate assemblies using kmer sketching in Dashing2 [55]. Dashing2 containment index was calculated to assess the pretransplantation overlap in the phageomes of wild donors and captive recipients.

To investigate the variation in phage or bacterial community composition between mouse groups, Principal Coordinate Analysis (PCoA) and Permutational Multivariate Analysis of Variance (PERMANOVA) were employed. PERMDISP2 [56] was used to test whether inter-individual variation in phageome or bacteriome composition varied between mouse groups. To identify phages that varied between mouse groups, mvabund R package [57] was employed.

To determine whether experimental phage transplantation altered phageome and bacteriome composition, we compared dissimilarities between microbiomes from each wild donor and the two recipient mice sampled at D-1, D2, and D7 posttransplantation with dissimilarities between all non-transplanted controls and all donors at the same time point. These comparisons were carried out using generalized linear mixed models (GLMMs) with a Gamma distribution, where the identity of the captive mouse and wild donor was modeled as a random factor. The same approach was used to assess changes in the proportion of temperate phages in transplanted and non-transplanted mice over the course of the experiment.

To investigate the posttransplantation changes in subcontig phage variability, we calculated Fst distances for each polymorphic position (SNP, i.e. Single Nucleotide Polymorphism) in each contig shared between the mice groups, indicating the extent of divergence between wild donors and transplanted mice or non-transplanted controls, using PoPoolation2 [58]. The Fst is a measure borrowed from population genetics that reflects the difference in SNP frequency between populations. For illustration: if there is a different variant at the same SNP in the population of wild donors versus captive recipients before transplantation, and if each of these variants occurs with a frequency of 1 in the respective group, then the Fst at this SNP between the two groups is 1. If the captive variant is replaced by the wild variant in the recipients after transplantation, the Fst at this SNP between the two groups drops to 0. Due to the bimodal distribution, the Fst values were categorized as low-Fst or high-Fst based on the median value and modeled as a binary response in GLMM with a binomial distribution. SNP identity was considered a random effect, and mouse strain, treatment group, day of the experiment, and their interactions were explanatory variables.

To identify phages potentially transmitted from donors to recipients, we searched for phage contigs present in wild donors and appearing in recipient mice after transplantation, but absent in the recipients before transplantation and in all non-transplanted controls throughout the experiment. We then calculated the frequency of simultaneous occurrence of these contigs in respective donor-recipient pairs and tested whether the true frequency was higher than expected by chance. This was achieved by randomly reshuffling the absence/presence data for each of the tested contigs across the donor samples (*n* = 1000 permutations) and recalculating randomized donor–recipient co-occurrences.

The temporal stability of phageomes and bacteriomes in captive mice was assessed using the average difference and bootstrap 95% confidence intervals between dissimilarities for the same or different individuals collected in two different phases of the experiment (D-1 vs. D2 or D2 vs. D7), excluding dissimilarities between mouse strains.

Correlations between bacteriomes and phageomes from the same individuals were assessed through Procrustes analysis, conducted separately for wild and captive populations and for different phases of the experiment. In addition to Procrustes analyses, we implemented a multilevel Sparse Partial Least Square (SPLS) regression for dominant phage contigs and bacterial ASVs [59]. Finally, utilizing GLMMs with a negative binomial distribution, we searched for phage contigs and bacterial ASVs whose abundances exhibited significant correlations. These analyses were focused on a subset of phage contigs and bacterial ASVs that contributed considerably to SPLS (loadings for one of the first two SPLS axes <0.2, *n* = 18 phage contigs and 12 ASVs).

The statistical analyses are described in more detail in Supplementary Methods.

## Results

### Characterization of mouse phageomes

**Figure 2:**
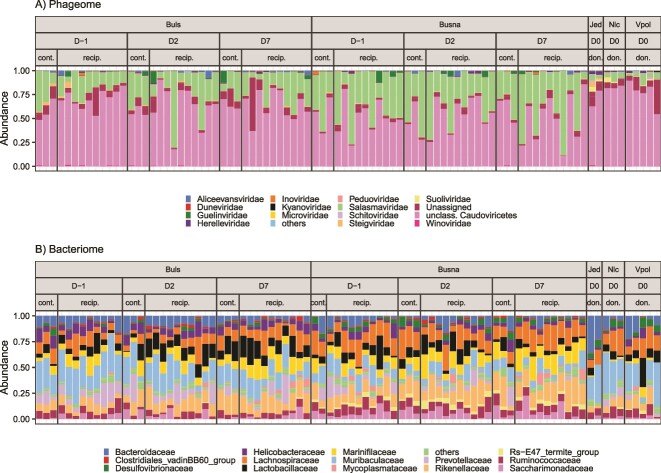
Proportions of (A) phage and (B) bacterial families detected in each sample; feces from two captive mouse strains (Buls and Busna) and from wild mice captured at three localities Jedov (Jed), Naloučany (Nlc), and Velké Pole (Vpol) were sampled prior to the experimental phageome transplantation, at Day-1 (D-1) or Day 0 (D0), and 2 days (D2) and 7 days (D7) after the transplantation; wild mice were donors of phage transplants (don.), and captive mice were either recipients (recip.) or non-transplanted controls (cont.).

A total of 13 754 phage contigs were identified in the meta-reference, with a median of 3610 phage contigs per sample (ranging from 2079 to 6191). The most abundant taxa in both wild and captive mice belonged to the class *Caudoviricetes* (Fig. 2) and included mainly the taxa that are not classified at the family level (60% of the total phageome) and the family *Salasmaviridae* (29%). A smaller portion of the phageome was represented by other *Caudoviricetes* families, and by ssDNA *Microviridae* and *Inoviridae* phages. However, the abundance of the latter two families cannot be accurately estimated due to the dsDNA-based preparation of sequencing libraries. Classification of phages based on capsid morphology (i.e. 2021 International Comittee on Taxonomy of Viruses [ICTV] taxonomy) revealed the dominance of podoviruses and siphoviruses, followed by myoviruses (Supplementary Fig. S1). A total of 6400 phages (46.5%), representing 27.1% of the phage community (per sample range = 6.3%–55.7%), were classified as temperate. The lifestyle of 17.3% phages accounting for 4.1% of the total phageome remained undetermined.

A re-analysis of the phageomes of SPF mice [32] showed a predominance of *Caudoviricetes* phages, which lack family-level taxonomy (73% of the total phageome). The most abundant phage family was *Suoliviridae* (order *Crassvirales*), comprising 26% of the phageome. The proportion of temperate phages was similar to that in non-SPF wild and captive mice: 51.3% of phage contigs, representing 26.1% of the phage community, were classified as temperate.

### Gut bacteriome is strong predictor of phageome

**Figure 3:**
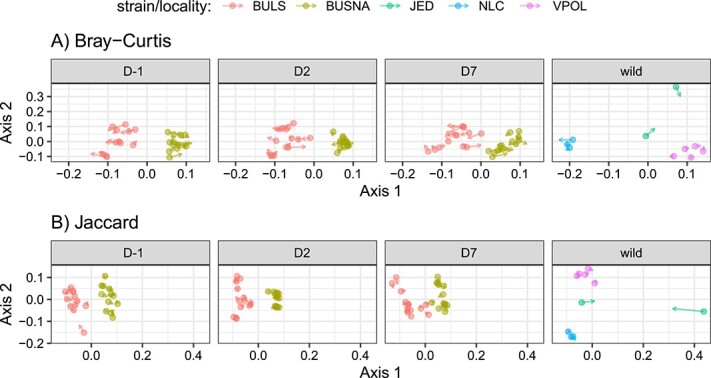
Procrustean superimposition of bacteriome and phageome distances between the samples; (A) Bray–Curtis and (B) Jaccard distances were superimposed separately for the microbiomes from wild mice (sampling localities JED, NLC, VPOL) and from captive mouse strains (BULS and BUSNA), sampled over the course of the experiment (D-1, D2, D7).

Composition of bacteriomes and phageomes was highly correlated with respect to the prevalence and abundance of bacterial ASVs and phage contigs in both wild and captive mice, as evidenced by Procrustes (Fig. 3, Supplementary Table S2) and multilevel SPLS analyses (Supplementary Fig. S2). Using a set of 13 phage contigs and 10 bacterial ASVs with considerable loading (<0.2) for either of the first two SPLS axes in captive mice, we identified eight significant co-occurrence links. These comprised mainly bacteria of the genera *Alistipes*, *Bacteroides* and unassigned *Prevotellaceae* and *Muribaculaceae* together with two family-level unclassified siphoviruses of the class *Caudoviricetes* and one *Salasmaviridae* phage (Supplementary Fig. S3).

According to host predictions, most phages interacted with dominant bacterial classes, e.g. *Lachnospiraceae*, *Oscillospiraceae*, and *Bacteroidaceae*. However, more than 50% of the phageome remained without a predicted host, even when using a relatively low iPHoP confidence score of 75 (Supplementary Fig. S4).

### Wild and captive mice have distinct phage communities

**Figure 4:**
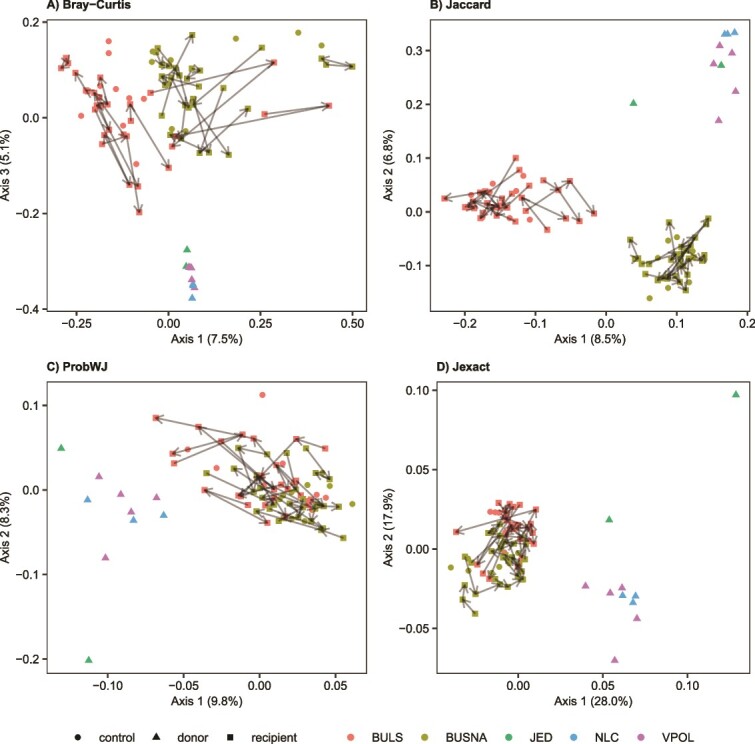
Principal coordinate analysis depicting variation in phageome composition measured as (A) Bray–Curtis, (B) Jaccard, (C) probWJ, and (D) Jexact dissimilarities between wild mice sampled at three localities: JED, VPOL, NLC and mice from two captive mouse strains: BUSNA and BULS; wild mice were donors of phage transplants, and captive mice were either recipients of phage transplants or non-transplanted controls; individual trajectories showing changes in the phageome of recipients from Day 0 to Day 7 of the experiment are indicated by arrows; wild and captive mice form distinct clusters throughout the experiment.

**Figure 5:**
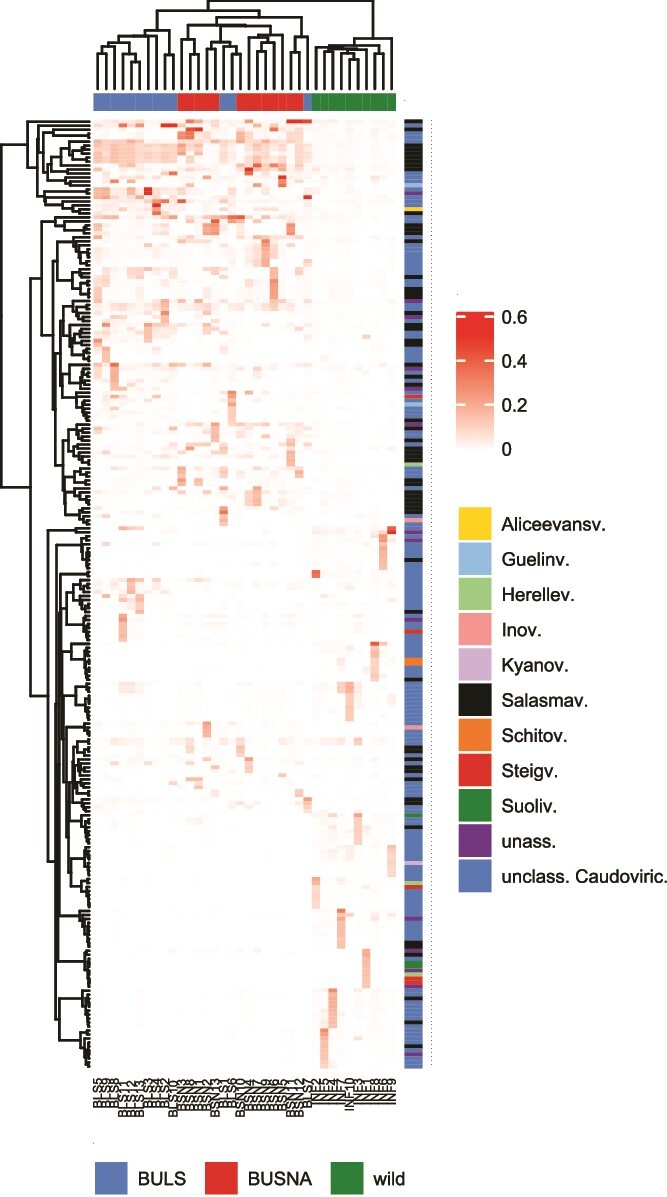
Cluster heatmap showing relative abundance of dominant phage contigs in mouse phageomes before the phage transplantation; phage contigs are clustered on the *y*-axis and colored by their taxonomic affiliation (family names in the legend are shortened, e.g. Myov. = *Myoviridae*); only contigs that were among the 10 most abundant in at least one sample were included, and their abundances were square rooted to suppress influence of extreme values; samples from wild mice and captive mice (BULS and BUSNA) form separate clusters on the *x*-axis. BUSNA and BULS samples are not separated, suggesting the role of rare phages in their differentiation; phageomes of wild mice show strong individual signatures represented by clusters of phage contigs with various taxonomy.

The phageomes of wild mice exhibited higher alpha diversity compared to those of captive non-SPF mice (Supplementary Fig. S5, Analysis of Variance [ANOVA]: *F*_2,32_ = 4.6, *P* = .0194 for contig richness and *F*_2,32_ = 8.6, *P* = .001 for Shannon diversity). The composition of wild and captive phageomes differed on all dissimilarity measures (Fig. 4, PERMANOVA *P* = .001, detailed in Supplementary Table S3), and significant variation was also found between sampling locations of wild mice (PERMANOVA *P* ≤ .002, detailed in Supplementary Table S3) and between the two captive mouse strains (PERMANOVA *P* = .001). Differential abundance analyses revealed higher relative abundance of *Suoliviridae* and *Schitoviridae* in wild then in captive mice, whereas the opposite was observed for *Salasmaviridae* (Supplementary Fig. S6). The phageomes of wild mice also showed higher interindividual variation on all dissimilarity measures except for Jaccard distance (PERMDISP2 *P* ≤ .001, detailed in Supplementary Table S4, Fig. 5).

The proportion of temperate phageome was increased in wild compared to captive mice (ANOVA: *F*_2,31_ = 9.7, *P* = .0005). The PCoA and PERMANOVA, where the relative abundances of phage contigs were binned according to family-level taxonomy of their putative hosts, suggested that wild mice harbor phages targeting a different spectrum of bacterial hosts than captive mice (pseudo-*F*_1,86_ = 7.6, *R*^2^ = 0.08, *P* = .001, Supplementary Fig. S7). Significant differentiation was revealed also for the two captive mouse strains (pseudo-*F*_1,76_ = 7.1, *R*^2^ = 0.09, *P* = .001).

### Phageome transplantation induces slight but significant shifts in recipient phageomes

The phageome transplantation from wild to captive mice did not result in significant changes in phageome alpha diversity, as shown by nonsignificant interaction between the treatment (i.e. transplanted individuals vs. controls) and day of experiment (Supplementary Table S5). Similarly, strong convergence of recipient to donor phageomes was not indicated by PCoA ordination, where the BULS and BUSNA phageomes remained in separate clusters throughout the experiment, distinct from the wild phageomes (Fig. 4).

**Figure 6:**
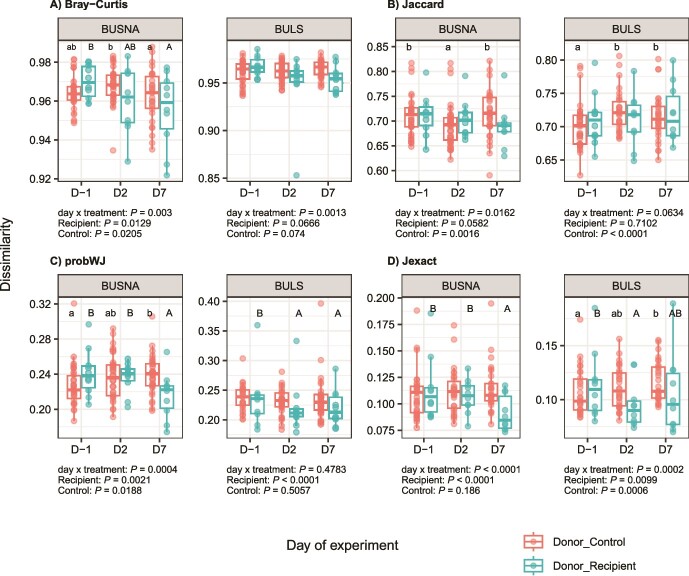
Differences in phageome composition between transplant donors and recipients (Donor_Recipient), or transplant donors and non-transplanted controls (Donor_Control), over the course of the experiment (D-1, D2, D7) measured as (A) Bray–Curtis, (B) Jaccard, (C) probWJ, and (D) Jexact dissimilarities; recipients/controls were mice from two inbred strains, BUSNA and BULS. Using GLMM, we first tested separately for each mouse strain whether the differences between recipients and controls in terms of their dissimilarity to wild donors changed over the course of the experiment (i.e. day × treatment interactions, *P* values are indicated below each plot); separate GLMMs were then applied to recipient and control mice (*P* values shown below each plot); if separate GLMMs for recipients and/or controls were significant, Tukey post hoc tests were performed, and the results were indicated in the corresponding plot by letter symbols (lower case for controls and upper case for recipients); a partial or complete overlap of letters across two boxplots (e.g. B–AB or A–A) means that these two groups do not differ; in the opposite case (e.g. A and B), the difference is statistically significant (*P* < .05 according to Tukey post hoc tests).

However, GLMM analyzes consistently provided evidence of slight but significant changes in phageomes caused by the transplantation. For all contig-level and kmer-based dissimilarity measures, the phageome dissimilarity of control and transplanted captive mice to wild donors changed over the course of the experiment (i.e. significant day × treatment interaction), and this change was often modulated by the mouse strain (i.e. significant day × treatment × strain interactions (Supplementary Table S6). To decipher these complex relationships, GLMMs were fitted separately for each mouse strain (Fig. 6). For BUSNA, the interaction between treatment level (i.e. controls vs. recipients) and the day of the experiment was significant for all types of dissimilarities, with control vs. transplanted mice generally showing greater divergence at later stages of the experiment. On the contrary, this interaction was significant only for Bray–Curtis dissimilarities in the case of BULS.

**Figure 7:**
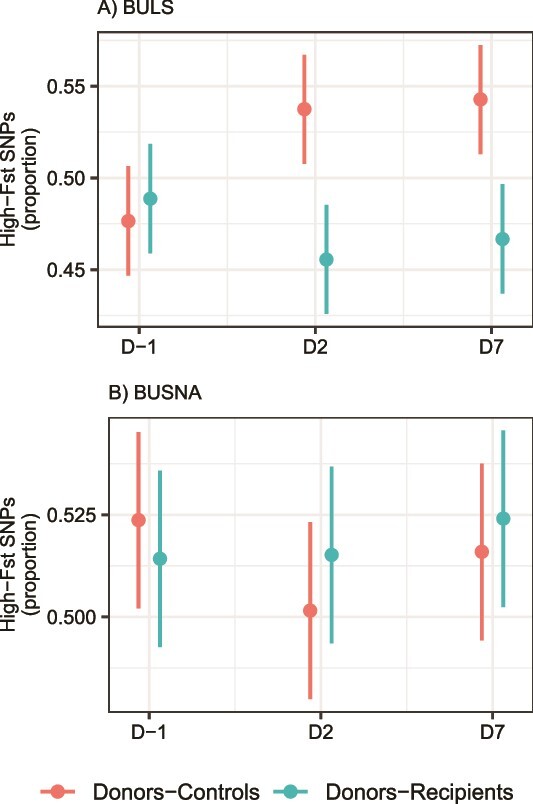
Changes in the subcontig phageome divergence in A) BULS and B) BUSNA mice between transplant donors and recipients (Donors–Recipients), or transplant donors and non-transplanted controls (Donors–Controls), over the course of the experiment (D-1, D2, D7), measured as the proportion of SNPs with high-Fst (i.e. above median).

The subcontig Fst-based differentiation between wild and captive phageomes also revealed significant interactions between strain, treatment, and the day of the experiment (GLMM: ΔDF = 2, χ^2^ = 26.621, *P* < .0001), indicating that the phageomes of captive mice exhibited strain-specific changes at the subcontig level after the transplantation. Separate analyzes for BULS indicated decreasing divergence between transplanted recipients and wild donors, while the opposite was true for non-transplanted controls (ΔDF = 2, χ^2^ = 32.295, *P* < .0001). In the case of BUSNA, transplanted mice and controls showed no differences (ΔDF = 2, χ^2^ = 3.104, *P* = .212), (Fig. 7). The overlap in phageome sequence content between wild donors and recipients before the transplantation was similar for both mouse strains (Dashing2 average containment index 0.48 and 0.44 for BUSNA and BULS, respectively).

Six-hundred-twenty-two phage contigs that were present in wild donors appeared in recipients after phage transplantation (but were absent in recipients before transplantation and in non-transplanted controls throughout the experiment). Potentially transmitted phages that were detected simultaneously in the respective donor-recipient pairs included 47.5% of these contigs (i.e. 296 contigs), which is significantly higher than the percentage resulting from the neutral expectation that these phages are randomly distributed in the donor cohort (~26.6%, Wilcoxon test for paired samples: *P* < .0001). Furthermore, 181 contigs (61.1% out of the potentially transmitted) occurred exclusively in recipients and their respective donors.

The temperate phages were underrepresented in the group of potentially transmitted contigs (*n* = 108, 36.5% of contigs) compared to the group of non-transmitted contigs (*n* = 6292, 46.7% of contigs, chi-squared test: ΔDF = 1, χ^2^ = 7.35, *P* = .0067). However, for the entire community, the proportions of temperate phages were not affected by phageome transplantation, as indicated by the nonsignificant day × treatment interaction (GLMM: ΔDF = 2, χ^2^ = 2.313, *P* = .3146) or day × treatment × strain interaction (ΔDF = 2, χ^2^ = 1.656, *P* = .4369).

### Gut bacteriome is not affected by phage transplantation

The bacteriome dataset included 1 827 644 high-quality sequences and 2518 ASVs (average per sample depth = 21 007; range = 10 728 – 29 528). Consistent with the phageome data, the composition of bacteriomes varied between captive and wild mice and between the sampling localities and inbred strains (Supplementary Fig. S8, Supplementary Table S3). Bacteriome of wild mice also exhibited greater interindividual variation than inbred strains (Supplementary Table S4).

Although the alpha diversity of captive bacteriomes decreased during the experiment (Supplementary Table S5), there were no differences between controls and transplanted mice, nor was the interaction between the treatment group and the sampling interval significant. The temporal stability of bacteriomes tended to be lower compared with the stability of phageomes, especially between D-1 and D2 (Supplementary Fig. S9).

Similarly as for phages, we tested whether bacteriome dissimilarity between recipients and donors decreased relatively to uninfected controls after the phage transplantation. However, the interaction between the day of the experiment and treatment was non-significant ΔDF = 2, χ^2^ = 4.721, *P* = .094 for Bray–Curtis and ΔDF =2, χ^2^ = 1.796, *P* = .407 for Jaccard dissimilarities). The same was true for the three-way interactions between the day of the experiment, the treatment, and the mouse strain, suggesting phageome transplantation did not affect bacteriome composition (ΔDF = 2, χ^2^ = 2.808, *P* = .246 for Bray–Curtis and ΔDF = 2, χ^2^ = 2.267, *P* = .322 for Jaccard dissimilarities, Supplementary Fig. S10).

## Discussion

### Phageomes of SPF laboratory mice vs. non-SPF mice from this experiment

The gut microbiota of SPF laboratory mice is aberrant in terms of the functions it provides to the superhost [31]. This has been attributed to the highly altered bacteriomes of SPF mice compared to their wild counterparts, connected to rederivation procedures and interventions to maintain the SPF state [60]. Whether the altered SPF bacteriome is coupled with an altered phageome is not known, because there has been no data on phageomes in non-SPF mice so far. We reanalyzed the data on phageomes of SPF mice from the study by Kim and Bae [32], and compared them with our non-SPF mice (i.e. captive mice from a conventional breeding facility and wild mice). We observed substantial differences in the taxonomic composition of the phageomes. In non-SPF mice, the *Salasmaviridae* family was the most abundant, comprising almost 30% of the phage community. In the SPF mice, the corresponding phageome fraction belonged to the crAss-like phages of the *Suoliviridae* family, and the *Salasmaviridae* accounted for <1%. In gut communities, these two families infect distinct host phyla: *Bacillota* and *Actinomycetota* in the case of *Salasmaviridae* [61], and *Bacteroidota* for *Suoliviridae* [62, 63]. Compared to the *Salasmaviridae* [64], the crAss-like phages are typical for their large genomes and complex biology [65, 66]. These taxonomic differences likely have unexplored functional consequences, as inferred from the major differences in phage–host interactions between these two groups of phages.

### Phageome differences between wild and captivity

To our knowledge, the comparative analyses of gut phageomes between wild and captive mammals are limited to a study on primates [30]. Consistent with this research, we observed systematic differences between the wild and captive house mouse phageomes, which involved community composition (Fig. 4, Supplementary Table S3), relative representation of phage taxa (Supplementary Fig. S6), and predicted bacterial hosts (Supplementary Fig. S7). The more variable environment and higher genetic diversity could explain the substantially greater differences in phageome composition between individuals in the wild (Supplementary Table S4) and their increased alpha diversity (Supplementary Fig. S5). The wild phageomes displayed strong individual signatures, evidenced by the sets of abundant phage contigs with low overlap with other mice (Fig. 5), a pattern resembling the phageomes of healthy adult humans [22].

In line with the phylogeographic structuring of some human phages [29], mouse phageomes were differentiated by sampling localities, possibly due to the structuring of the superhost population and/or locality-specific environmental factors (Supplementary Table S3). The phageomes of the two mouse strains living in the same environment were clearly separated, indicating vertical transmission or superhost dependent selective filtering of phages from the environmental pool [20, 67].

Previous research has shown that ecological conditions, particularly diet composition and its variations, can affect the induction of prophages from lysogenic gut bacteria [19, 25, 32]. Increased intensity of such prophage induction agents in the wild could explain the higher proportion of temperate phages in the lytic cycle in wild mice compared to captive mice. On the other hand, possible differences in the frequency of lysogeny between the bacteriomes of captive and wild mice could also contribute to this pattern.

### Interactions between phages and bacteria

The structuring of phageomes and bacteriomes mirrored each other in terms of community composition (i.e. differences between wild and captive mice and between sampling locations and captive mouse strains) and inter-individual variability (i.e. increased variability in wild mice compared to captive mice) (Supplementary Tables S3 and S4). The connection between bacteriome and phageome was also evident from the strong correlation between these two components of the gut microbiota at the individual level (Fig. 3). Co-occurrence analysis revealed eight significant phage-bacteria links involving six different bacterial ASVs and three different phage contigs (Supplementary Fig. S3), supporting the finding of Kim and Bae [32], that mouse gut phages tend to be generalists rather than specialists. Six of these co-occurrences were positive, and two were negative, suggesting complex dynamics of phage-bacteria interactions in the house mouse gut. Two of the phage contigs were assigned as temperate *Caudoviricetes* siphophages, and their predicted hosts matched the taxonomy of the co-occurring bacterial ASVs at genus (*Alistipes*) or order (*Bacteroidales*) level. This suggests that the five positive co-occurrences detected might reflect the induction of prophages from the respective bacteria. The remaining virulent phage of the *Salasmaviridae* family co-occurred with 2 bacterial ASVs, both of which were implicated in the interaction with one of the siphophages, but the direction of the co-occurrences was opposite. Furthermore, the predicted host of this phage (phylum *Bacillota*) was taxonomically distant from the interacting ASVs, indicating that co-occurrence analysis captured secondary shifts in the bacterial community composition rather than a direct phage–bacteria interaction.

### Experimental transplantation of wild phageomes to captive mice

The transplantation of gut phageomes has gained increasing attention, with studies focusing on the beneficial regulatory effects of phage transplants on experimentally disrupted or diseased microbiota in animal models [37–40, 68] or humans [35, 69]. Despite some inconsistencies, most of these studies report posttransplantation changes in phageome structure or diversity, the incorporation of donor phages into recipient communities, and associated changes in gut bacteriomes. In our experiment conducted on healthy adult mice, the phageomes of recipients did not show any abrupt changes, such as shifts in alpha diversity, an increased proportion of temperate phages, or substantial compositional divergence. However, the distances between the phageomes of donors and recipients decreased (Fig. 6, Fig. 7) and wild phages appeared in the captive mice after the transplantation, indicating transfer of wild phages into the captive microbiomes. Overall, this suggests that orally administered non-native phages can invade adult and healthy mouse phageomes, without replacing or destabilizing them.

A previous study [34] on fecal microbiota transplantation, which involves phages along with gut bacteria, reported a preferential transfer of temperate phages, while another study [70] showed that virulent phages are transferred as well. In our experiment, the putative transferred fraction was enriched in virulent phages. This could be connected to the absence of bacteria carrying prophages in the transplanted filtrates. On the other hand, it may also reflect an increased ability of virulent phages to infect non-native bacterial populations.

The convergence of phageomes from recipients to wild donors depended on the phage resolution level in a superhost-dependent manner. The phageomes of the BUSNA strain consistently converged at the contig-level phage resolution, whereas this signal was weaker in BULS (Fig. 6). However, BULS phageomes clearly converged to the wild donors when considering subcontig variability (Fig. 7), implying that wild phages integrated into BULS phageomes often assembled into the same contigs as resident phages. It is likely that this was not due to higher initial similarity of BULS and wild phageomes as the overlap in phageome sequence content between wild donors and captive recipients before the transplantation (i.e. containment index) was slightly lower for BULS than for BUSNA. It thus seems that different phages integrated into the phageome of each mouse strain, and that either the resident microbial communities or the superhost itself determined which subset of the available phages was integrated. In line with the study on *Clostridium difficile* infection (CDI) patients [70], this finding suggests the context-dependence of the outcomes of the phageome transplantation.

Changes in recipients’ gut bacteriome composition occurred primarily during the first 2 days posttransplantation, while the communities were more stable in the subsequent 5 days (Supplementary Fig. S9). This pattern was common to both recipient mice and non-transplanted controls, and thus likely unconnected to disruption of the bacteriome structure due to the transfer of wild phages. Moreover, the change in bacteriomes preceded the change in phageomes, which remained relatively stable in the first 2 days compared to the next 5 days. Similarly, we did not detect convergence of recipient bacteriomes to the bacteriomes of wild donors (Supplementary Fig. S10). This result may not be surprising, given the resilience of phage communities to the invasion of wild phages and the strong correlation between the composition of the two gut microbiota components throughout the experiment. Nevertheless, we cannot exclude that bacteriome changes followed changes in the phageomes with a time-delay that was beyond the scope of our experiment. Other factors, such as administration of only a single dose of the phageome transplant could also play a role [68]. Overall, it appears that the transplantation of wild phages did not induce any detectable effects in the captive bacteriomes and that changes in captive bacteriomes during the experiment were likely due to other factors, such as the handling stress.

## Conclusions

We characterized the composition and interaction between gut bacteriomes and phageomes in a non-primate vertebrate outside of SPF settings. We found a pronounced divergence in phageome composition between house mice with distinct ecologies, represented by two captive-bred strains and wild living mice. The most abundant phage family in these non-SPF mice was *Salasmaviridae*, in contrast to SPF mice in which a similar proportion of the phageome was occupied by crAss-like phages from the *Suoliviridae* family. At the individual level, the gut bacteriome proved to be a strong predictor of phageome composition. Experimental transplantation resulted in the transfer of wild phages to captive phageomes, and the phages transferred differed between the two mouse strains. However, the transfer did not induce major changes in the structure of phage communities in either mouse strain, nor did it have any measurable effects on their gut bacteriome. This suggests that the adult healthy gut microbiota is robust to this type of experimental intervention, providing baseline information for follow-up research on the safety of phage allotransplantation.

## Supplementary Material

Supplementary_Figures_wrae178

Supplementary_Methods_wrae178

Supplementary_Tables_wrae178

## Data Availability

16S rRNA gene amplicon and metagenomic sequencing data are archived in the European Nucleotide Archive (project accession numbers: PRJEB76455 and PRJEB76457). Sample metadata, including individual ENA accessions, are available in the online Supplementary Table S1. The scripts for the data analyses presented in this article have been deposited in the GitHub repository (https://github.com/jakubkreisinger/HM_phages).
