# Supplementary material for: Convergence of gut phage communities but not bacterial communities following wild mouse bacteriophage transplantation into captive house mice
Source: ISME J. 2024 Sep 14;18(1):wrae178. doi: 10.1093/ismejo/wrae178 (PMC11440513; doi:10.1093/ismejo/wrae178)
Supplement: Supplementary_Figures_wrae178 [file supplementary_figures_wrae178.pdf]

Supplementary figures to:

Convergence of gut phageomes but not bacteriomes after experimental transplantation of wild mouse bacteriophages into captive house mice.

Dagmar Čížková[1]*, Pavel Payne[1,2]*, Anna Bryjová[1], Ľudovít Ďureje[1], Jaroslav Piálek[1], Jakub Kreisinger[2]

1 Institute of Vertebrate Biology of the Czech Academy of Sciences, Brno, Czech Republic
2 Department of Zoology, Faculty of Science, Charles University, Prague, Czech Republic

Supplementary Figure S1

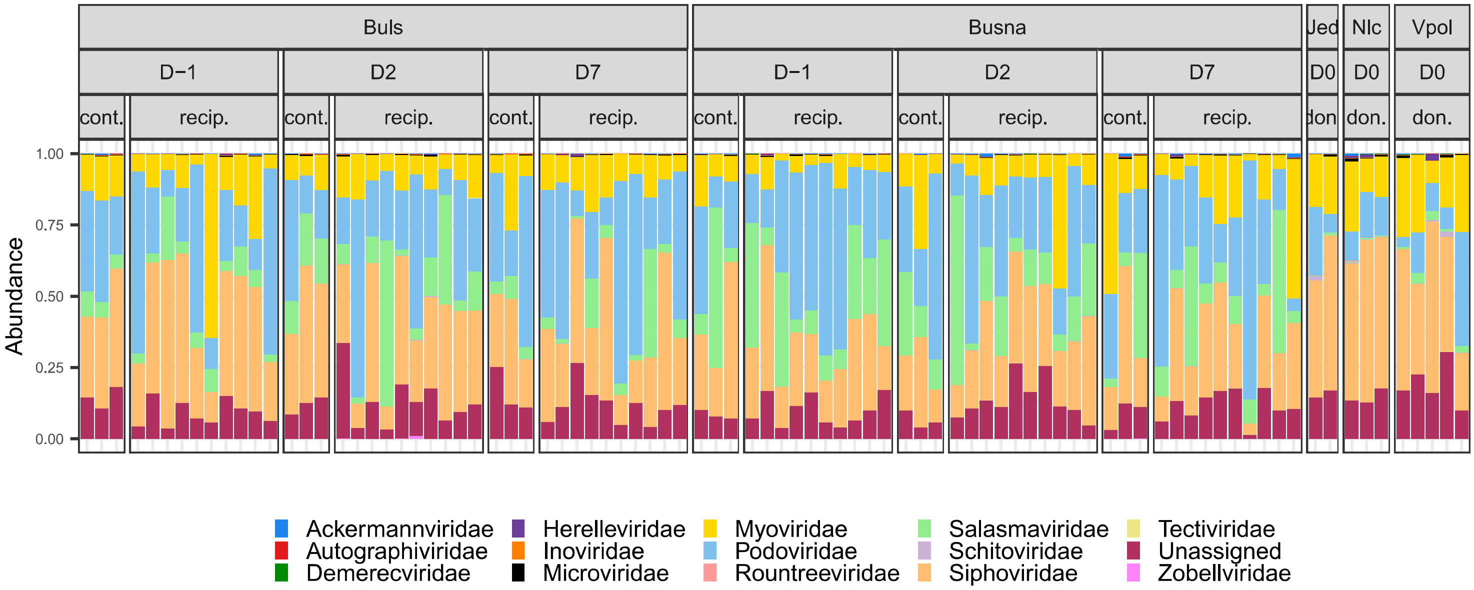

Figure S1: Proportions of phage families detected in each sample, according to 2021 ICTV taxonomy, that reflects capsid morphology. Feces from two captive mouse strains (Buls and Busna) and from wild mice captured at three localities Jedov (Jed), Naloučany (Nlc), and Velké Pole (Vpol) were sampled prior to the experimental phageome transplantation, at day -1 (D-1) or day 0 (D0), and 2 days (D2) and 7 days (D7) after the transplantation. Wild mice were donors of phage transplants (don.) and captive mice were either recipients (recip.) or non-transplanted controls (cont.).

Supplementary Figure S2

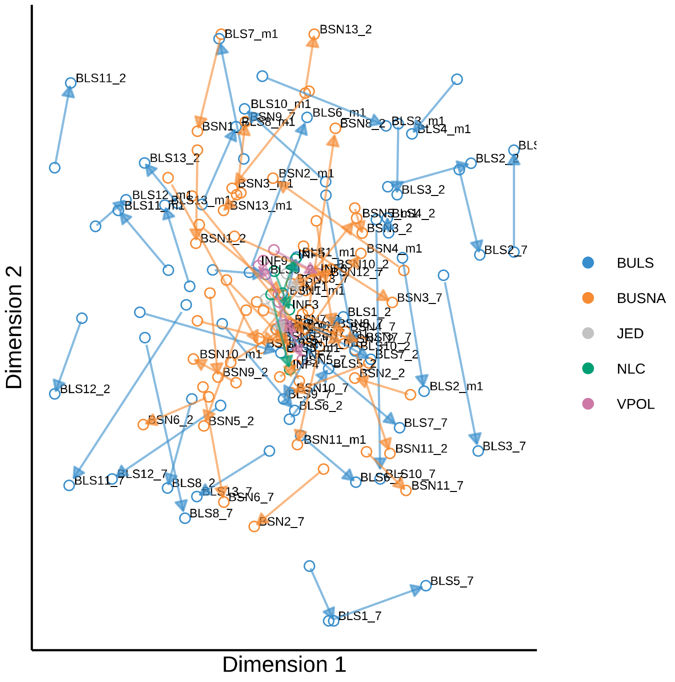

Figure S2: Arrow plot showing concordance between bacteriome and phageome profiles according to Sparse Partial Least Square Regression. Colors distinguish mice from three wild sampling localities (JED, NLC, VPOL) and from two inbred strains (BULS and BUSNA).

Supplementary Figure S3

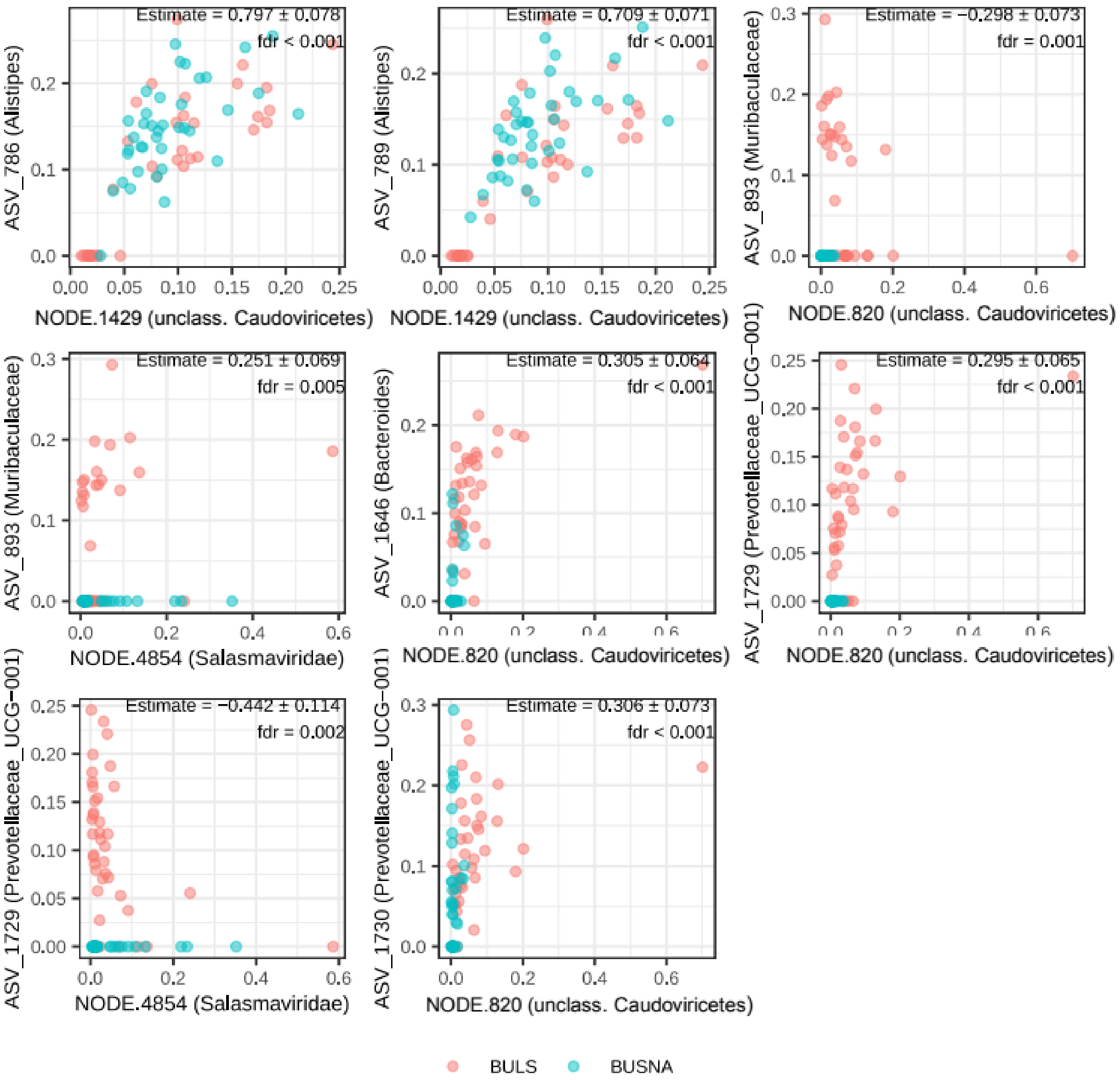

Figure S3: Scatterplots showing relative abundances of bacterial ASVs and phage contigs that were significantly correlated according to GLMMs in captive mice. Values were squareroot transformed for the visualization purposes. Estimates of GLMM regression parameter, corresponding standard errors and false discovery rates corrections are shown. These analyzes were not done for wild mice because of small sample size.

Supplementary Figure S4

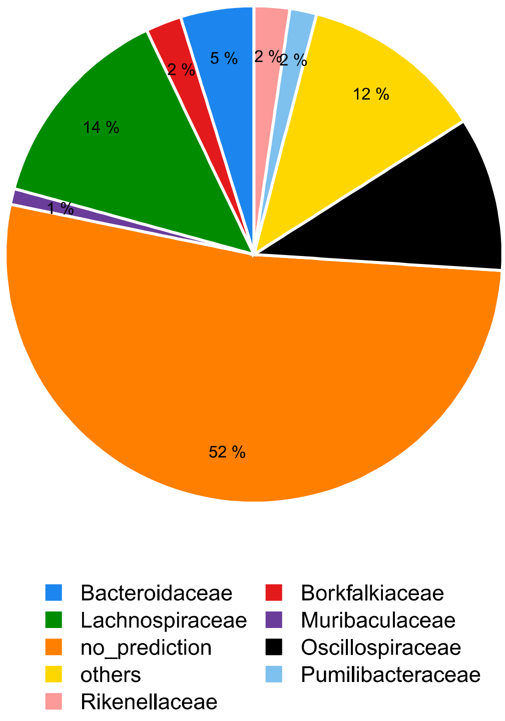

Figure S4: Relative representation of phages targeting different bacterial hosts (family-level taxonomy is shown), based on the iPHoP host predictions.

Supplementary Figure S5

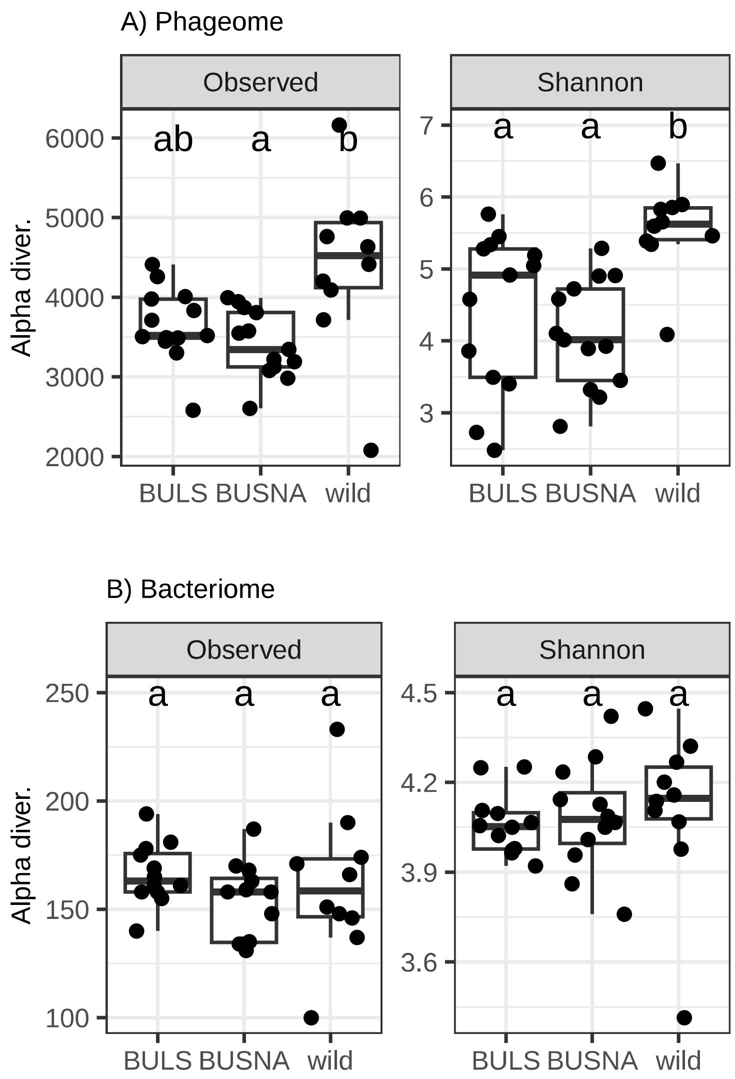

Figure S5: Variation of alpha diversity in A) phageome and B) bacteriome between wild mice and two laboratory mouse strains (BULS and BUSNA) during the pre-transplantation phase of the experiment. Alpha diversity was assessed as the observed number of phage contigs or bacterial ASVs (Observed) and their Shannon diversity (Shannon). Different letters above boxplots indicate significant differences between groups. A partial or complete overlap of letters across two boxplots (e.g. ab–a or a-a) means that these two groups do not differ. In the opposite case (i.e. a-b), the difference is statistically significant ($p < 0.05$ according to Tukey post-hoc tests).

Supplementary Figure S6

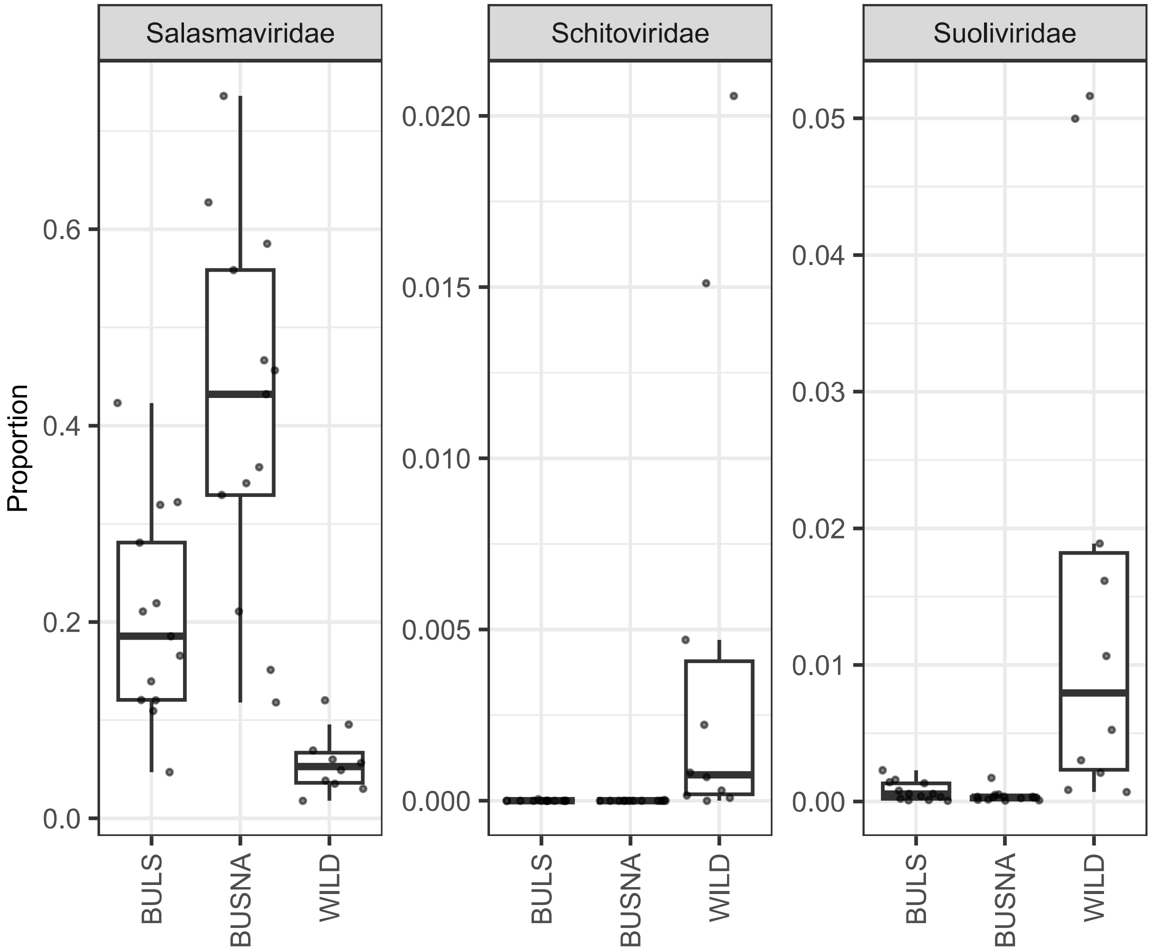

Figure S6: Bacteriophage families, whose relative abundance varied between mice groups (i.e. BUSNA and BULS captive mouse strains, and wild mice), according to differential abundance analysis.

Supplementary Figure S7

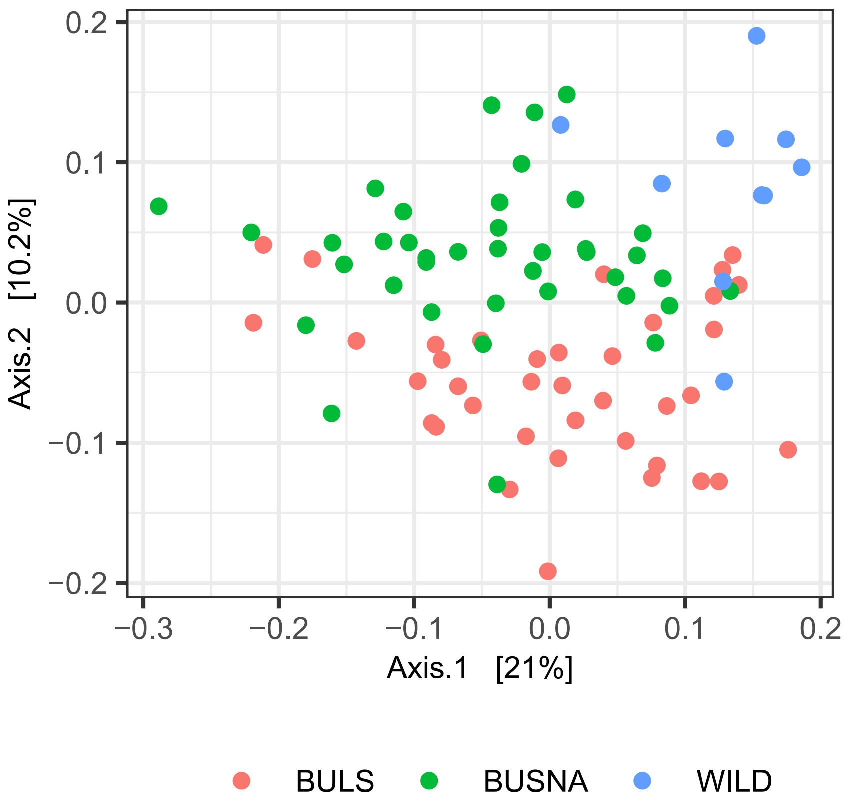

Figure S7: PCoA plot indicating that phageomes of wild (WILD) and captive mice (BUSNA and BULS strains) differ by the spectrum of predicted bacterial hosts.The analysis was performed on Bray-Curtis dissimilarities.

Supplementary Figure S8

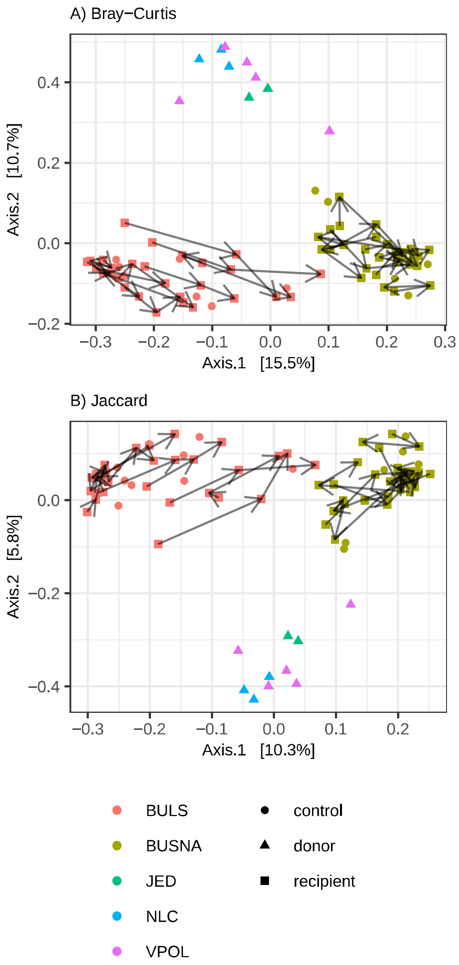

Figure S8: Principal Coordinate Analysis depicting variation in bacteriome composition measured as A) Bray-Curtis, B) Jaccard dissimilarities between wild mice sampled at three localities: JED, VPOL, NLC and mice from two captive mouse strains: BUSNA and BULS. Wild mice were donors of phage transplants and captive mice were either recipients of phage transplants or non-transplanted controls. Individual trajectories indicating changes in the phageome of recipients from day 0 to day 7 of the expriment are indicated by arrows.

Supplementary Figure S9

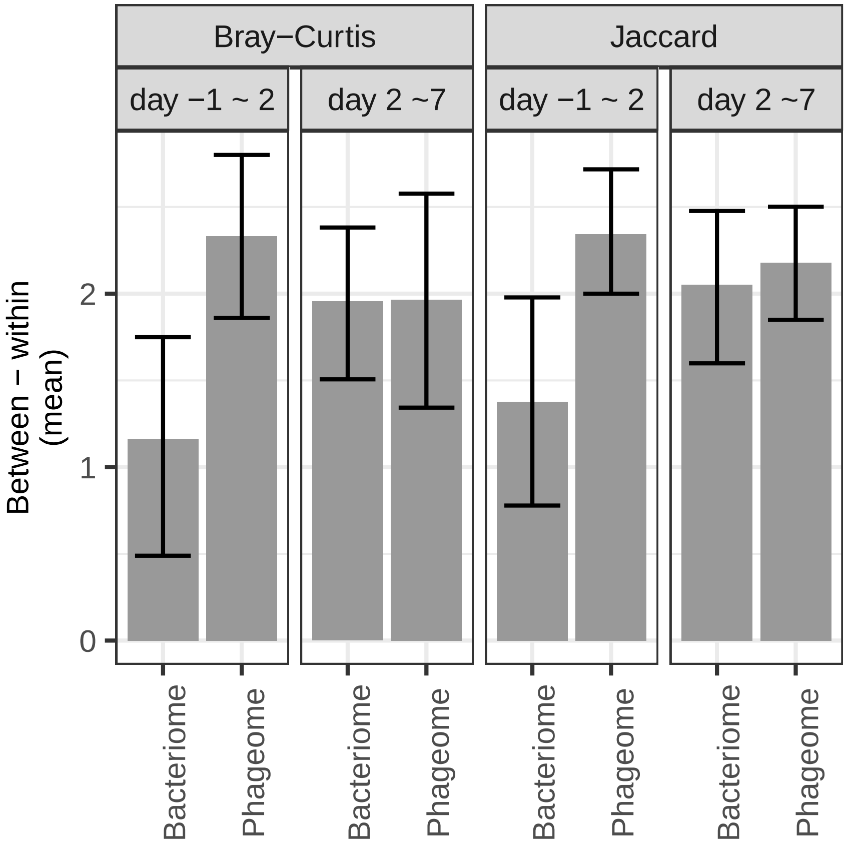

Figure S9: Temporal stability of bacteriome and phageome in captive mice. The stability was assessed as the average difference between standardized Bray-Curtis or Jaccard dissimilarities for the same or different individuals collected at two different phases of the experiment (D-1 vs. D2 or D2 vs. D7). Dissimilarities between mouse strains were not considered in these analyzes. Error bars correspond to 95% bootstrap-based confidence intervals, so their overlap indicates a lack of statistical significance.

Supplementary Figure S10

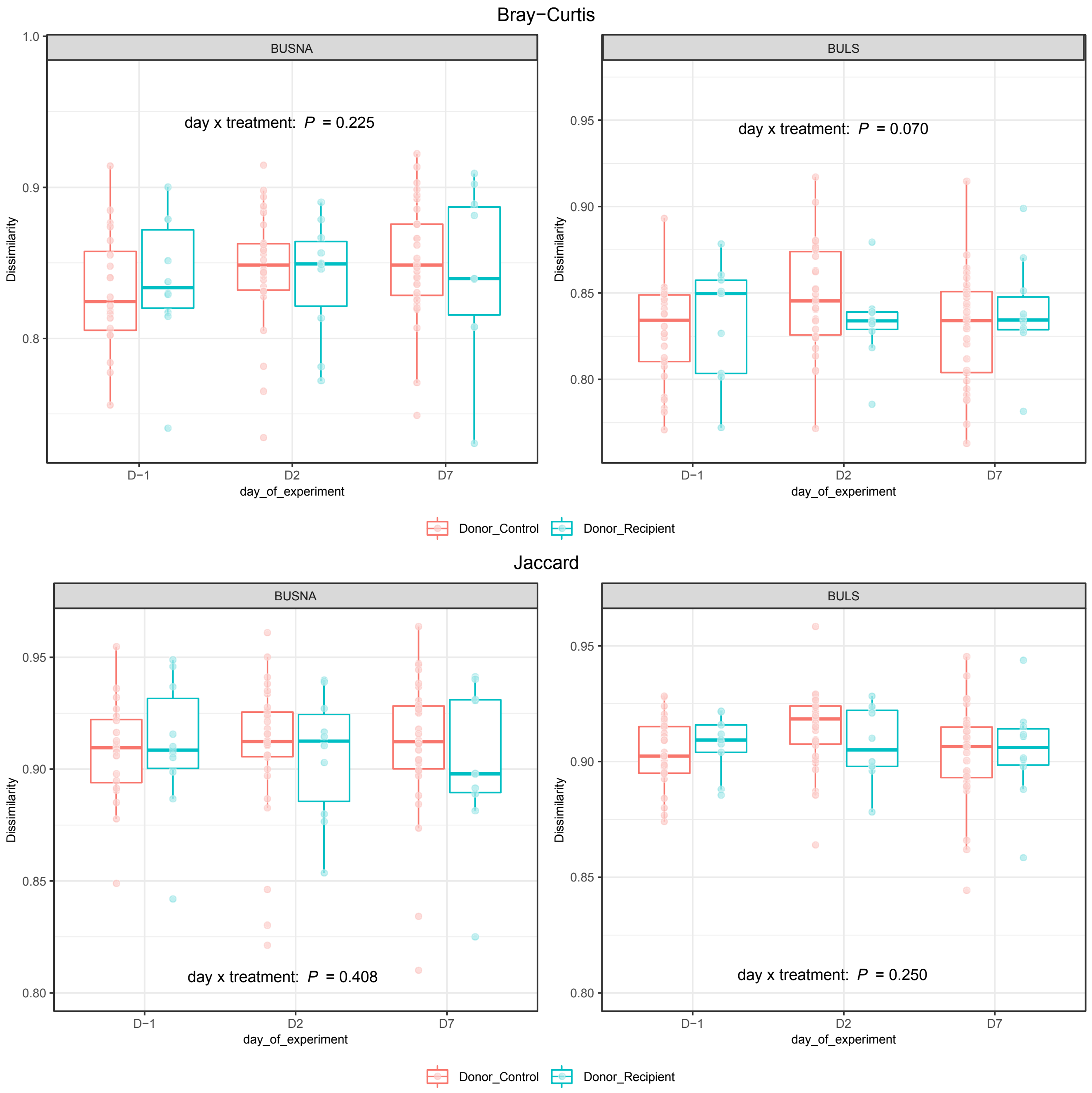

Figure S10: A) Bray-Curtis, B) Jaccard baceriome dissimilarities between transplant donors and recipients (Donor_Recipient), or transplant donors and non-transplanted controls (Donor_Control), over the course of the experiment (D-1, D2, D7). Recipients/controls were mice from two inbred strains, BUSNA and BULS. Using GLMM, we first tested separately for each mouse strain whether differences between recipients and control in terms of their dissimilarity with wild donors varied over the course of the experiment (i.e. day x treatment interaction, associated *P* values shown). Because the interaction was not significant for any mouse strain and dissimilarity index, we did not apply separate GLMMs for recipient and control mice.
