# Supplementary material for: Convergence of gut phage communities but not bacterial communities following wild mouse bacteriophage transplantation into captive house mice
Source: ISME J. 2024 Sep 14;18(1):wrae178. doi: 10.1093/ismejo/wrae178 (PMC11440513; doi:10.1093/ismejo/wrae178)
Supplement: Supplementary_Methods_wrae178 [file supplementary_methods_wrae178.pdf]

Supplementary Methods to:

Convergence of gut phageomes but not bacteriomes after experimental transplantation of wild mouse bacteriophages into captive house mice.

Dagmar Čížková[1,2], Pavel Payne[1,2], Anna Bryjová[1], Ľudovít Ďureje[1], Jaroslav Piálek[1], Jakub Kreisinger[2]

1 Institute of Vertebrate Biology of the Czech Academy of Sciences, Brno, Czech Republic
2 Department of Zoology, Faculty of Science, Charles University, Prague, Czech Republic

Methods:

Bacteriome profiling
We used dada2 (Callahan *et al.*, 2016) for quality filtering (reads with > 2 expected error were eliminated) and denoising of fastq files. The resulting Amplicon Sequence Variants (hereafter ASV) were checked for the presence of chimeric variants using uchime (Edgar *et al.*, 2011) and only those that were detected in both technical duplicates were retained (e.g., Pafčo *et al.*, 2018). Taxonomic assignment was based on dada2 implementation of the RDP classifier (Wang *et al.*, 2007); posterior confidence threshold set to 80%) and the Silva reference database version 132 (Quast *et al.*, 2013).

Phageome profiling
The 88 sequencing libraries encompassed total of 622 M read pairs (min. = 2.5 M, max. = 22.8 M and median = 5.7 M read pairs per library). Raw reads were filtered and trimmed using Skewer 0.2.2 (Jiang *et al.*, 2014) and assembled with metaSPAdes 3.14 (Nurk *et al.*, 2017), using two complementary strategies to check the consistency of the results. First, reads from all samples were assembled together to construct a meta-reference. Second, separate assemblies for individual samples were constructed.
Despite the sequenced metagenomes were enriched for virus-like particles some contamination with non-viral DNA is still expected. Therefore, we filtered the assemblies for the contigs of presumably phage origin by combining three approaches and contigs identified as viruses/bacteriophages by at least one approach were pre-selected: (1) Contigs longer than 4 kbp were analysed with Marvel 0.2 (Amgarten *et al.*, 2018), which predicts dsDNA viruses (particularly Caudovirales) based on their genomic features (gene density, strand shifts) and fraction of genes with hits to pVOGs hmm profiles, using default settings and significance thresholds. (2) Contigs longer than 3 kbp were analysed with Cenote-Taker (Tisza *et al.*, 2021), which predicts viral contigs by searching for hallmark virus genes. We used the Unlimited Breadsticks module with the settings recommended for VLP enriched metagenomes. (3) All contigs longer than 0.5 kbp were classified using Blobtools v1.1.1 (Laetsch and Blaxter, 2017), based on the blast hits to NCBI nt database and to UniProt reference proteomes database and those assigned to a bacteriophage order were selected.
The pre-selected contigs were classified to the Class and Family taxonomic level with Demovir (https://github.com/feargalr/Demovir), using Diamond 2.0.8 (Buchfink *et al.*, 2015) searches against the Viruses subset of the UniProt reference proteomes database (release 2024_02). The contigs assigned by Demovir to non-phage viruses as well as T7 contigs (i.e the spiked in controls; complete T7 genomes were assembled for each sample) were further

filtered out, creating the final sets of phage contigs. CheckV was used to estimate the quality of phage genomes (Nayfach et al. 2021).
In the next step, the sequencing reads were mapped back to the meta-reference and to the separate assemblies using Bowtie2 2.3.2 (Langmead and Salzberg, 2012). Using SAMtools v1.11 (Li *et al.*, 2009), we retrieved the reads from individual samples, which mapped to phage contigs. Resulting matrix with abundances of phage contigs across individual samples, was subsequently used for statistical analyses.
Lifestyle of the phages (virulent vs. temperate) was predicted using PhaTYP, a tool that exhibits high accuracy even for short fragments of phage genomes (Shang er al,. 2023).
Bacterial hosts of the phages were predicted with iPHoP v1.3.3 software (Roux et al., 2023) using minimum confidence score 75.
Phageome sequences from 6 samples (ERR2059953-ERR2059956, ERR2059965-ERR2059967) of specific pathogen free (SPF) laboratory mice generated by (Kim and Bae, 2018) were downloaded from ENA database and analysed using the bioinformatics pipeline described above. The authors (Kim and Bae, 2018) mention problem with the over-ampflicication of small circular genomes of Microviridae phages that likely occured during the whole-genome amplification step of the metagenomic DNA from the filtrates. Therefore we excluded 11 Microviridae contigs from our analyses.

Statistical analyses
We used analysis of variance (ANOVA) and linear mixed models (LMM) to analyze the variation in alpha diversity between mouse groups and treatment levels. Shannon diversity and contig/ASV richness were used as response variables, with the number of variants detected log10-transformed to achieve a normal distribution of model residuals. In the case of LMM, the identity of the individual was included as a random effect to account for pseudoreplications arising from repeated sampling of the same mouse.

The analyzes of bacteriome and phageome composition were mainly based on the dissimilarity of these communities between samples. First, we calculated binary Jaccard dissimilarities, accounting for the presence/absence of phage contigs or bacterial ASVs, and Bray-Curtis dissimilarities, accounting for the relative abundances of contigs/ASVs. Jaccard dissimilarities were calculated after the rarefaction of abundance matrices. For bacteria, the abundance matrices were constructed based on the number of reads corresponding to each ASV in each sample. For phages, the matrices were obtained by mapping the sequencing reads to the meta-reference, as described above.
For phageomes, we also calculated community dissimilarities based on their sequence content, using the individually assembled samples. This was done by kmer sketching in Dashing2 (Baker and Langmead, 2019), using two types of distances, analogous to Jaccard and Bray-Curtis measures. “Jexact” which reflects only the presence/absence of each unique kmer was computed on phage contig sequences, by exact sketching using full m-mer sets (--set). “ProbWJ” which weights the occurrence of each unique kmer was computed on the reads mapped to phage contigs, using ProbMinHash sketching (--prob). For all analyses we used kmer length of 31 (-k31), the sketch size of 26 (--sketch-size-l2 26). For “ProbWJ” distances (employing the sequencing reads) the minimum occurrence of a unique kmer was set to 2 (--count-threshold 2). The results were outputted as mash distances (--mash-distance).
In addition, we assessed the initial (pre-transplantation) overlap in the phageomes of wild donors and captive recipients, as the proportion of kmers common to a donor and a recipient to all recipient’s kmers (i.e. Dashing2 containment index; with the same settings as for “Jexact”).

The dissimilarity measures were used to analyze variation in phage or bacterial community composition between mouse groups, using Principal Coordinate Analysis (PCoA) and PERMANOVA (i.e., adonis function from the R package vegan; Oksanen *et al.*, 2022). In addition, cluster heatmap was constructed to show the variation in the relative abundance of dominant phage contigs (the ten most abundant contigs in at least one sample). Contig abundances were square rooted to suppress influence of extreme values.Phage families that varied between mouse groups we identified using mvabund R package (Wang *et al.*, 2012). PERMDISP2 (i.e., betadisper function from the R package vegan; Oksanen *et al.*, 2022) was used to test whether inter-individual variation in phageome or bacteriome composition varied between mouse groups.
Finally, to assess whether the phageomes differ in the spectrum of host bacteria, we calculated Bray-Curtis dissimilarity from relative abundances of phage contigs binned according to the family (or higher) taxonomy of their putative bacterial hosts (based on iPHoP host predictions mentioned above) and applied PCoA and PERMANOVA as described above.

To determine whether the phageome transplantation resulted in changes in recipients' phageome composition and whether these changes were reflected in bacteriome variation, we extracted bacteriome and phageome dissimilarity values between each wild donor and the two recipient mice sampled at pre-transplantation day D-1, and post-transplantation days D2 and D7, as well as dissimilarities between all non-transplanted controls and all donors. The resulting vector of dissimilarities was used as a response variable in generalized linear mixed models (GLMMs) with Gamma distribution. The GLMMs were designed to test whether the dissimilarity to donor changed between individual stages of the experiment in a different way for control vs phageome-transpalnted captive mice (i.e., interaction treatment level x day of experiment) and whether the response to the transplantation varied between the two mouse strains (i.e., interaction treatment level x day of experiment x strain). The identity of the captive mouse and wild donor was modeled as a random factor to account for pseudoreplications of the data.

To check the post-transplantation changes of the sub-contig phage variability, we calculated Fst distances for each site in each contig that was shared between the mice groups (i.e. the extent of divergence between wild donors and transplanted recipients or non-transplanted controls) using Popoolation2 (Kofler et al. 2011). First, individual bam files (mapping of reads to the meta-reference) from the same mice group were merged and variants between the groups were summarized with SAMtools mpileup, followed by Popoolation mpileup2sync and fst-sliding.pl (--min-count 2 and –min-coverage 4). Variation was estimated using a mixed model in which SNP identity was included as a random effect. Because of the bimodal distribution of Fst values, which prevented us from using a Gaussian or other standard continuous distribution, we categorized the Fst values as low or high according to the median value and modeled them as a binary response. Similar to the contig-level analyzes, mouse strain, treatment group, day of experiment, and their interactions were considered as explanatory variables.

We also looked for phage contigs that were present in wild donors and appeared in the recipient mice after transplantation but were not detected in the pre-transplantation phase and were also absent in all non-transplanted controls at each stage of the experiment. We then calculated the frequency of simultaneous occurrence of these contigs in respective donor-recipient pairs and tested whether the true frequency was higher than expected by chance. To do this, we randomly reshuffled the absence/presence data for each of the tested

contigs across the donor samples (n = 1000 permutations) and recalculated randomized donor-recipient co-occurrences.

The temporal stability of phage and bacterial community for captive mice was assessed using the average difference and corresponding 95% bootstrap-based confidence intervals between dissimilarities for the same or different individuals collected in two different phases of the experiment (D-1 vs. D2 or D2 vs. D7), excluding dissimilarities between different mouse strains. To obtain comparable results for phageomes and bacteriomes, dissimilarity values were standardized by centering and dividing the (centered) dissimilarities by their standard deviation.
Correlation between bacteriomes and phageomes from the same individuals was assessed by Procrustean analysis performed separately for data from the wild population and from different phases of the experiment. To account for the fact that bacteriomes and phageomes may vary systematically between mouse strains and locations, which may lead to false rejection of the null hypothesis, we defined strains or locations as blocks (i.e., "strata") and performed permutations within these blocks during statistical testing.
In addition to Procrustean analyzes, we fitted a multilevel Sparse Partial Least Square (SPLS) regression for dominant phage contigs and bacterial ASVs (i.e., represented by > 0.5% of reads, n = 33 and 44, respectively) using the R package mixOmics (Rohart *et al.*, 2017), specifying the identity of the strain or locality along with the individual identity in the corresponding design matrix.
Finally, using GLMMs, we searched for phage contigs and bacterial ASVs whose abundances showed significant correlation. These analyzes focused on a subset of phage contigs and bacterial ASVs that contributed considerably to SPLS (loadings for one of the first two SPLS axes < 0.2, n = 18 phage contigs and 12 ASVs). For all these combinations, we fitted GLMMs with negative binomial distributions, considering the focal ASV read counts in each sample as the response, the log-transformed proportions of focal phage contig as the explanatory variable, and the log-transformed bacterial read counts in each sample as the model offset. Finally, false discovery rates (FDR; Benjamini and Hochberg, 1995) were used as a correction method for multiple testing.

Phage genome quality
Based on the CheckV predictions of viral genome quality, the phage contigs included 1% of complete genomes (6% of total phageome), 2% of high-quality genomes (4% of total phageome), 3% of medium-quality genomes (20% of total phageome), 66% of low-quality genomes (63% of total phageome) and 29% of genomes remained unpredicted (7% of total phageome):

Parameter	Subset	Complete genome	High quality	Medium quality	Low quality	Not determined
% of contigs	all phages	1.06	1.56	2.91	65.92	28.54
% of community	all phages	6.06	4.40	19.84	62.69	7.01
	wild mice phages	4.72	5.89	10.41	69.94	9.04
	Buls mice phages	7.47	4.90	18.68	60.65	8.31
	Busna mice phages	5.00	3.51	23.43	62.89	5.18

Interestingly, the low-quality flag was widely distributed among genomes within the typical dsDNA phage genome size (44% of 200kb - 16kb viral contigs) and those with high

coverage (69% of genomes with more than 100x SPAdes k-mer coverage, corresponding to approximately 800x per base coverage).
Given that highly covered and long contigs are unlikely to arise from fragmented assemblies, we hypothesized that CheckV predictions might not work optimally on our dataset. This has already been pointed out by Dutilh et al., (2021): “Because of its dependency on reference genomes, CheckV performs less well with viral genomes that have few or distant known relatives and when terminal repeats are lacking from the contig.” Another possibility is that these contigs could be fragments of longer non-viral genomes highly abundant in the dataset.
To investigate further, we selected contigs flagged as low-quality by CheckV but were between 50kb-16kb in size and had SPAdes k-mer coverage higher than 100 for additional inspection. We performed structural and functional annotation of these genomes using PhageScope (https://phagescope.deepomics.org/). Although many of the predicted genes were unknown, we found multiple homologues of known phage proteins in each of these contigs (see the figure “Gene content of contigs” below), suggesting they indeed represent phages, albeit relatively distant from genomes in the databases.
We also provide an annotated genome map of the first contig from the set (see the figure “Genome map” below). This contig most likely represents a closed genome rather than a fragment: both ends of the contig contain the same Pfam domain of the tail collar protein, indicating either an opening point of a circular assembly or a genome with terminal redundancy.
Therefore, it seems evident that at least some genomes from our dataset were assigned to the low-quality category due to the lack of closely related genomes in CheckV databases, rather than being genome fragments or non-viral genomes. Practically, this means that for the CheckV low-quality genomes (which constitute the majority of the dataset), we cannot reliably determine if they are high-quality genomes with distant relatives in the databases or genome fragments.

Figure Gene_content: PhageScope predicted gene content of phage contigs with high coverage and length corresponding to phage genomes, that were assesed as low quality genomes by checkV.

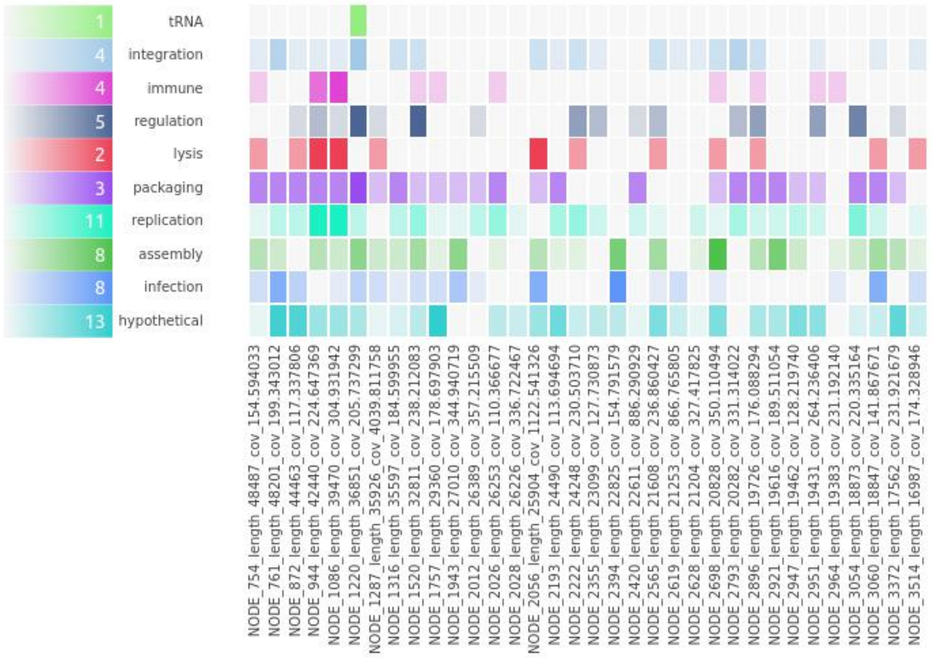

Figure Genome_map: An example of a PhageScope annotated map of a genome which was among the phage contigs with high coverage and length corresponding to phage genomes, that were assesed as low quality genomes by checkV.

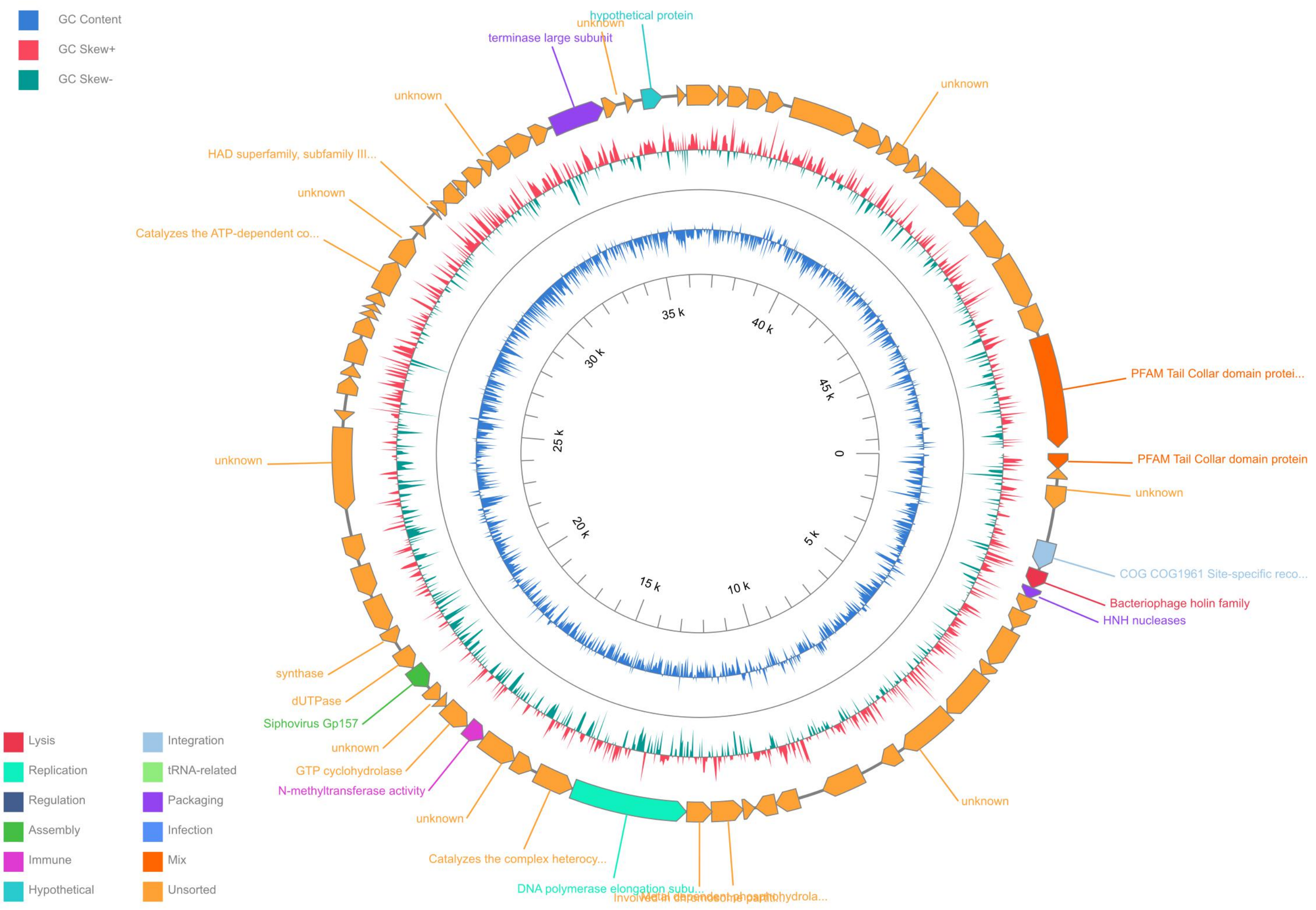

References:

Amgarten D, Braga LPP, da Silva AM, Setubal JC (2018). MARVEL, a Tool for Prediction of Bacteriophage Sequences in Metagenomic Bins. *Front Genet* **9**: 304.

Benjamini Y, Hochberg Y (1995). Controlling the False Discovery Rate: A Practical and Powerful Approach to Multiple Testing. *Journal of the Royal Statistical Society Series B (Methodological)* **57**: 289–300.

Buchfink B, Xie C, Huson DH (2015). Fast and sensitive protein alignment using DIAMOND. *Nat Methods* **12**: 59–60.

Callahan BJ, McMurdie PJ, Rosen MJ, Han AW, Johnson AJA, Holmes SP (2016). DADA2: High resolution sample inference from Illumina amplicon data. *Nat Methods* **13**: 581–583.

Clooney AG, Sutton TDS, Shkoporov AN, Holohan RK, Daly KM, O'Regan O, *et al.* (2019). Whole-Virome Analysis Sheds Light on Viral Dark Matter in Inflammatory Bowel Disease. *Cell Host Microbe* **26**: 764-778.e5.

Dutilh BE, Cassman N, McNair K, Sanchez SE, Silva GGZ, Boling L, et al. A highly abundant bacteriophage discovered in the unknown sequences of human faecal metagenomes. Nat Commun 2014; 5: 4498.

Edgar RC, Haas BJ, Clemente JC, Quince C, Knight R (2011). UCHIME improves sensitivity and speed of chimera detection. *Bioinformatics* **27**: 2194–2200.

Jiang H, Lei R, Ding S-W, Zhu S (2014). Skewer: a fast and accurate adapter trimmer for next-generation sequencing paired-end reads. *BMC Bioinformatics* **15**: 182.

Kim M-S, Bae J-W (2018). Lysogeny is prevalent and widely distributed in the murine gut microbiota. ISME J 12: 1127–1141.

Laetsch DR, Blaxter ML (2017). BlobTools: Interrogation of genome assemblies. *F1000Res* **6**: 1287.

Langmead B, Salzberg SL (2012). Fast gapped-read alignment with Bowtie 2. *Nat Methods* **9**: 357–359.

Li H, Handsaker B, Wysoker A, Fennell T, Ruan J, Homer N, *et al.* (2009). The Sequence Alignment/Map format and SAMtools. *Bioinformatics* **25**: 2078–2079.

Nayfach S, Camargo AP, Schulz F, Eloe-Fadrosh E, Roux S, Kyrpides NC. CheckV assesses the quality and completeness of metagenome-assembled viral genomes. *Nat Biotechnol* 2021; 39: 578–585.

Nurk S, Meleshko D, Korobeynikov A, Pevzner PA (2017). metaSPAdes: a new versatile metagenomic assembler. *Genome Res* **27**: 824–834.

Oksanen J, Simpson GL, Blanchet FG, Kindt R, Legendre P, Minchin PR, *et al.* (2022). vegan: Community Ecology Package.

Pafčo B, Čížková D, Kreisinger J, Hasegawa H, Vallo P, Shutt K, *et al.* (2018). Metabarcoding analysis of strongylid nematode diversity in two sympatric primate species. *Sci Rep* **8**.

Quast C, Pruesse E, Yilmaz P, Gerken J, Schweer T, Yarza P, *et al.* (2013). The SILVA ribosomal RNA gene database project: improved data processing and web-based tools. *Nucleic Acids Res* **41**: D590–D596.

Rohart F, Gautier B, Singh A, Cao K-AL (2017). mixOmics: An R package for ‘omics feature selection and multiple data integration. *PLOS Computational Biology* **13**: e1005752.

Roux S, Camargo AP, Coutinho FH, Dabdoub SM, Dutilh BE, Nayfach S, *et al.* (2023). iPHoP: An integrated machine learning framework to maximize host prediction for metagenome-derived viruses of archaea and bacteria. *PLOS Biology* **21**: e3002083.

Shang J, Tang X, Sun Y. PhaTYP: predicting the lifestyle for bacteriophages using BERT. Briefings in Bioinformatics 2023; 24: bbac487.

Tisza MJ, Belford AK, Domínguez-Huerta G, Bolduc B, Buck CB (2021). Cenote-Taker 2 democratizes virus discovery and sequence annotation. *Virus Evolution* **7**: veaa100.

Wang Q, Garrity GM, Tiedje JM, Cole JR (2007). Naïve Bayesian Classifier for Rapid Assignment of rRNA Sequences into the New Bacterial Taxonomy. *Appl Environ Microbiol* **73**: 5261–5267.

Wang Y, Naumann U, Wright ST, Warton DI (2012). mvabund– an R package for model-based analysis of multivariate abundance data. *Methods in Ecology and Evolution* **3**: 471–474.
